# Surgery Performed Under Propofol Anesthesia Induces Cognitive Impairment and Amyloid Pathology in ApoE4 Knock-In Mouse Model

**DOI:** 10.3389/fnagi.2021.658860

**Published:** 2021-04-26

**Authors:** Jong-Ho Kim, Harry Jung, Yeonkyeong Lee, Jong-Hee Sohn

**Affiliations:** ^1^Department of Anesthesiology and Pain Medicine, Chuncheon Sacred Heart Hospital, Hallym University College of Medicine, Chuncheon, South Korea; ^2^Institute of New Frontier Research, College of Medicine, Hallym University, Chuncheon, South Korea; ^3^Department of Neurology, Chuncheon Sacred Heart Hospital, Hallym University College of Medicine, Chuncheon, South Korea

**Keywords:** post operative cognitive dysfunction, anesthesia, surgery, Alzheimer’s disease, propofol

## Abstract

**Background:** Postoperative cognitive dysfunction (POCD) following anesthesia and surgery is a common and severe complication, especially in elderly patients. A pre-existing cognitive impairment may impart susceptibility to further cognitive dysfunction; the mechanism remains unclear. We hypothesized that the specific impacts of anesthesia and surgery on individuals with preclinical Alzheimer’s disease (AD) may render them more susceptible to an increase in the risk of cognitive impairment. The aim of this study was to compare the cognitive impairment between normal adult mice and those with preclinical AD after propofol anesthesia and surgery.

**Methods:** We performed abdominal surgery in cognitively pre-symptomatic, 5-month-old male mice with sporadic AD (apolipoprotein E4 allele, ApoE4-KI) and age-matched (C57BL/6J) controls. Propofol anesthesia (170 mg/kg) was induced *via* retro-orbital injection over 2 h. Morris water maze (MWM) and Y-maze tests were conducted 2 days before and 2, 4, and 7 days after surgery. The mean escape latencies and spontaneous alternation percentages were the major outcomes. Neuronal apoptosis in hippocampal sections was evaluated using the terminal dUTP nick-end labeling (TUNEL) assay. Hippocampal amyloid beta (Aβ) levels were assessed *via* quantitative immunohistochemistry (IHC).

**Results:** The control mice exhibited increased mean escape latencies of MWM at postoperative 2 and 4, but not at day 7; ApoE4-KI mice exhibited such increases at postoperative days 2, 4 and 7. Significant differences between ApoE4-KI and control mice in terms of the mean escape latencies were evident at days 2 and 7 (both *P* < 0.05). However, performance on a non-hippocampal memory tasks (Y-maze test) did not differ. More TUNEL-positive neurons were evident in the hippocampal CA3 region of ApoE4-KI mice at postoperative days 2 and 4, but not at day 7 compared to the control group (both *P* < 0.05). IHC revealed significantly elevated Aβ deposition in the hippocampal CA3 region of ApoE4-KI mice at postoperative days 4 and 7 compared to control mice (both *P* < 0.05).

**Conclusions:** Propofol anesthesia followed by surgery induced persistent changes in cognition, and pathological hippocampal changes in pre-symptomatic, but vulnerable AD mice. It would be appropriate to explore whether preclinical AD patients are more vulnerable to POCD development.

## Introduction

Post-operative cognitive dysfunction (POCD) after anesthesia and surgery is a common severe complication, especially in elderly patients. POCD is characterized by a loss of attention, memory impairment, and personality changes that persist for months or years (Qiao et al., 2015). The 1-week and 3-month incidences of POCD in surgical patients aged over 60 years are 25.8–41.4% and 9.9–12.7% respectively (Moller et al., 1998; Monk et al., 2008). POCD has been associated with various adverse outcomes, including impaired recovery and increased morbidity and mortality (Saczynski et al., 2012). POCD imposes a substantial healthcare burden, including prolonged hospital stays and increased healthcare costs (Lundström et al., 2005). Thus, a better understanding of the relationship between cognitive impairment, anesthesia and surgery is critical.

However, does POCD really exist? What are the diagnostic criteria? The question of whether surgery or anesthesia is associated with either an earlier onset of dementia or a higher risk of dementia, remains? To date, the terminology used for cognitive classification of general populations has also been employed when investigating cognitive changes after anesthesia and surgery. Recently, a nomenclature working group has recommended, based on the DSM-5 criteria, that “perioperative neurocognitive disorders” should serve as an overarching descriptor of cognitive impairment evident either pre- or post-operatively, including cognitive decline after an acute event (post-operative delirium) and such a decline, diagnosed up to 30 days after procedure (delayed neurocognitive recovery) and also up to 12 months (post-operative neurocognitive disorder). The POCD research criteria used in previous studies may meet the DSM-5 criteria for mild and major neurocognitive disorders (Evered et al., 2018).

Although POCD generally resolves within 1 year of surgery, it may persist for several years and increases the incidence of dementia (Moller et al., 1998; Abildstrom et al., 2000; Chen et al., 2014). Several epidemiological studies have suggested that anesthesia and certain surgical procedures may increase the AD risk. One study found that cumulative general and spinal anesthesia prior to the age of 50 years was inversely correlated with age at AD onset (Breteler et al., 1991; Bohnen N. et al., 1994; Gasparini et al., 2002; Zuo and Zuo, 2010; Seitz et al., 2011). However, other clinical reports found no association between anesthesia and AD (Bohnen N. I. et al., 1994; Seitz et al., 2011). Anesthetics may precipitate or exacerbate neurocognitive disorders including AD (Baranov et al., 2009). Inhaled anesthetics triggered amyloid beta (Aβ) and tau accumulations in a mouse model of AD (Run et al., 2009; Perucho et al., 2010). A previous study suggested that patients with AD neuropathology identified on cerebrospinal fluid (CSF) analysis (even in the absence of clinically detectable AD symptoms) may be susceptible to POCD (Evered et al., 2016). However, the pathogenesis of POCD has not been completely elucidated. POCD development is associated with surgical trauma and general anesthesia (Terrando et al., 2011). Although retrospective human studies have suggested a link between surgery and the cognitive outcome, many questions remain unanswered. Animal studies have shown that both anesthesia and an operation *per se* independently affect cognition; however, the effect of surgery may predominate (Eckenhoff and Laudansky, 2013). Both surgery and anesthesia have been implicated in the development of POCD in a vulnerable brain such as that of the Alzheimer transgenic mouse model (Mardini et al., 2017). Previous findings implied that AD or a similar underlying pathology may render individuals more susceptible to the potential neurotoxic effects of surgical stress and/or anesthesia, and increase the risk of cognitive impairment progression (Belrose and Noppens, 2019).

Most AD patients (~98%) have late-onset sporadic AD; however, most animal models currently used to develop AD therapies are based on familial AD mutations in *APP*, *PSEN1* or *PSEN2*. Although AD has multiple causes, the human apolipoprotein E4 allele (ApoE4) is the strongest genetic risk factor. This increases the risk of AD development and decreases the age of onset (Corder et al., 1993; Liu et al., 2013). The ApoE4 transgenic knock-in (KI) mouse is an animal model of sporadic AD. AD development is slow and progressive, with a pre-symptomatic course of several years to decades. Aβ accumulation has been suggested to cause AD. Gradual Aβ accumulation commences 10–20 years before the occurrence of dementia symptoms in at least one-third of individuals at risk of AD (Bennett et al., 2006; Villemagne et al., 2013; Dubois et al., 2016). When these markers are present in cognitively normal individuals, the condition is termed preclinical AD (Sperling et al., 2011). ApoE4-KI mice exhibit age-dependent impairments in hippocampus-dependent learning and memory commencing at 15–18 or 12 months (Bour et al., 2008; Leung et al., 2012; Yin et al., 2014). Whether mice that are genetically vulnerable, such as ApoE4 KI preclinical mice, are at risk of development of cognitive impairment or dementia after surgery has not yet been completely elucidated.

We hypothesized that the specific impacts of anesthesia and surgery on patients with preclinical AD might render them more susceptible to progression of cognitive impairment and accelerated AD. We tested this hypothesis using the vulnerable brain of the preclinical ApoE-KI mouse model. We compared the cognitive impairments and pathological changes in adult control and preclinical AD mice after propofol-induced general anesthesia and surgery.

## Materials and Methods

### Experimental Animals

**Figure 1:**
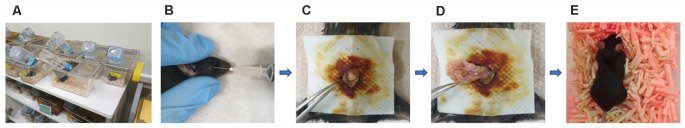
Experimental animals, anesthesia, and the surgical method. **(A)** Animals were housed with food and water available Alzheimer’s disease (AD) *ad libitum* in the hallym clinical translational research center. **(B)** General anesthesia was induced with 170 mg/kg propofol delivered *via* retro-orbital injection; the total duration of anesthesia was up to 2 h. **(C)** Surgery commenced with creation of a median abdominal incision approximately 1.5 cm in length in the peritoneal cavity. **(D)** The viscera were then gently manipulated. **(E)** After surgery, the mice were placed on heated pads and were returned to their cages after recovery.

This study was approved by the institutional review board of Hallym University (no. Hallym R2 2019-9, Chuncheon, South Korea). Male Apoe^tm1.1(APOE*4)Adiuj^ (ApoE4-KI) mice (*n* = 20) and male C57BL/6J (B6) mice (*n* = 20) were obtained from Central Laboratory Animal Incorporation (Seoul, South Korea). The mice were 5 months old at the time of anesthesia and surgery. At this age, ApoE4-KI mice exhibit underlying progressive Alzheimer neuropathology, but their cognitive function is normal (Grootendorst et al., 2005). They thus have preclinical AD. The animals were housed under a 12-h/12-h light/dark cycle (light on at 08:00) with free access to food and water (Figure 1A). The laboratory was maintained at 23 ± 2°C and 55 ± 10% humidity. The study followed the guidelines of the institutional Animal Care and Use Committee of Hallym University.

### Experimental Timeline

We formed eight groups of five mice each (B6 pre-operative and post-operative 2, 4, and 7 days vs. ApoE4 KI pre-operative and post-operative 2, 4, and 7 days). Before the experiment, all mice underwent water-maze training twice daily with inter-trial intervals of 4 h over 5 days. Two days before propofol anesthesia and surgery, all mice performed the Y-maze test and then the water-maze test with an inter-test interval of 60 min. The pre-operative groups (B6, *n* = 5/ApoE4 KI, *n* = 5) were sacrificed with perfusion. Two days later, the remaining mice were anesthetized with propofol and subjected to surgery. Two days after surgery, all mice performed the Y-maze test and then the water- maze test with an inter-test interval of 60 min. In the three groups (post-operative 2, 4, and 7 days), only five mice from the 2 day group were sacrificed. The experiment was conducted in the same timeline on both the 4 and 7 day groups.

### Anesthesia and Surgery

B6 and ApoE4-KI mice were anesthetized with 170 mg/kg propofol (Provine % [w/v], Baxter, Gujarat, India) delivered *via* retro-orbital injection (Figure 1B). The induction dose of propofol was 50 mg/kg and to maintain general anesthesia, 10 mg/kg was injected six times (every 10 min). The onset of anesthesia was marked by a reduced righting reflex and loss of the pedal withdrawal reflex. After induction, the pedal withdrawal reflex was tested 5 min. The absence of a response to a noxious stimulation was determined by a negative toe pinch response. We noninvasively monitored the blood pressure of the tail artery (BP-98A; Softron, Tokyo, Japan) and oxygen saturation (SpO_2_, percutaneous oxygen saturation). The body temperature was maintained at 37°C at all times. The total anesthesia duration was up to 2 h. After shaving the surgical site and sterilizing it with povidone-iodine solution, a median abdominal incision (approximately 1.5 cm) was made in the peritoneal cavity (Figure 1C), followed by insertion of a sterile probe and gentle manipulation of the viscera for 3 min (Figure 1D). Sterile 4–0 silk thread sutures were then used to close the peritoneal lining and the skin. All surgeries were performed by a single operator; each procedure required 10 min. After surgery, the mice were placed on heated pads and, after recovery, were returned to their cages (Figure 1E).

### Behavioral Testing

#### The Morris Water Maze Test

The Morris water maze (MWM) test was used to assess spatial learning and memory. Each mouse performed five acquisition trials (maximal swimming time 120 s; 5 s on the platform; 4 h inter-trial interval per day) over five consecutive days. The MWM test was performed before (2 days) and after (2, 4, and 7 days) surgery. Each mouse was placed in a circular pool (diameter 100 cm) filled with water (21–22°C) that was rendered opaque by addition of white paint. The mouse was trained to find a hidden platform (diameter 8 cm) submerged 1 cm below the water surface. Visual cues were placed on the four walls of the pool at 0.5 m distance from the center. All movements were video-tracked (NoldusEthoVision XT, Leesburg, VA, USA). We recorded swimming speeds, the times spent in the four quadrants, swim path lengths, and escape latencies. The mean escape latency time was the absolute escape latency and the ratio of this to the preoperative figure was the relative escape latency.

#### The Y-Maze Test

The Y-maze test was used to assess spatial and short-term memory. Each mouse was placed in a white Y-maze (the three arms were designated A, B and C). Each arm was 40 × 12 × 10 cm (length × height × width) in dimension. Each mouse was placed at the end of one arm and allowed to move freely through the maze for 5 min. All arm and center-point entries were video-tracked. Alternation behavior was defined as consecutive entries into all the three arms. The major outcome was spontaneous alternation (alternation index = alternations/maximum alternations *100, %). The mean alternation percentage was the absolute spontaneous alternation and the relative spontaneous alternation, the ratio thereof to the preoperative figure the spontaneous alternation.

### Histological Analysis

#### Hematoxylin and Eosin Stain

Mice were sacrificed 2 days pre-operatively and 2, 4 and 7 days post-operatively. Brain tissues were perfused after the behavioral tests (MWM and Y-maze) and fixed for 1 day in 4% (v/v) paraformaldehyde. The tissues were immersed in 30% (w/v) sucrose solution and completely embedded in optimal cutting temperature (OCT) compound prior to cryostat sectioning (10 μm). Hematoxylin-and-eosin (H&E) staining revealed hippocampal morphology and size.

#### Terminal-Deoxynucleotidyl Transferase Mediated Nick End Labeling (TUNEL) Assay

*In situ* detection of DNA fragments was performed using the Dead End Fluorometric terminal-deoxynucleotidyl transferase mediated nick end labeling (TUNEL) system (Promega, Madison, WI, USA). The sections were permeabilized with 0.2% (v/v) Triton X-100 for 5 min at room temperature. After washing, the sections were exposed to a mixture of the rTdT enzyme and equilibration buffer for 1 h at 37°C and the chamber was then covered with aluminum foil to protect against reaction to direct light. The reaction was stopped by placing the slices in a jar containing TdT Stop Buffer for 15 min. After washing in 1× phosphate-buffered saline (1× PBS), the slices were counterstained with 1 μg/ml 4′,6-diamidino-2-phenylindole (Molecular Probes, Eugene, OR, USA), incubated for another 10 min; and rinsed in 1× PBS three times for 5 min each time. Positive cells were counted under a fluorescence scanning microscope (Olympus, Tokyo, Japan) by an operator blinded to the experimental design. Eight coronal sections at the level of the hippocampus were examined in each of the ApoE4-KI and control B6 brains. The total number of TdT-labeled cells was counted in each section.

#### Immunohistochemistry (IHC)

Five animals from each group were deeply anesthetized with 2.2% (v/v) isoflurane (Hana Pharm, Company Limited, Gyeonggi-Do, South Korea) and transcardially perfused using normal saline with simultaneous exsanguination from the right atrium, followed by perfusion with 4% (v/v) paraformaldehyde in 0.1 mol/L phosphate buffer (pH 7.4) for 24 h after MWM and Y-maze testing. Brain tissues were incubated at 4°C overnight in 4% (v/v) paraformaldehyde. Fixed brain tissues were blocked into OCT. Frozen tissue blocks were sectioned (10 μm) using a cryostat microtome (Leica, Wetzlar, Germany). The slices were incubated in 0.3% (v/v) hydrogen peroxide for 15 min to quench endogenous peroxidase activity and then blocked with 2% (v/v) horse serum for 60 min. The sections were next incubated in a solution of a primary antibody against Aβ 1–42 (1:100, ab10148; Abcam, Cambridge, UK) overnight at 4°C and washed at room temperature, incubated with HRP-linked secondary antibody (1:250; Thermo Fisher Scientific Inc., Waltham, MA, USA) for 60 min, and washed again. HRP activity was detected using 3,3′-diaminobenzidine as the substrate. Tissues were washed three times for 5 min each time in 1× PBS and then counterstained with Mayer’s hematoxylin. Finally, the stained hippocampal tissues were observed under a light microscope (Carl Zeiss Microscopy GmbH, Zeiss, Germany). Positive cells were counted using ImageJ software (ImageJ 1.49v, National Institutes of Health, Bethesda, MD USA).

### Biochemical Analysis

Serum acetylcholine levels were measured using a mouse acetylcholine ELISA kit (MBS733116, MyBioSource Limited, USA) following the manufacturer’s protocol. Serum samples were prepared from whole blood *via* centrifugation at 3,000 rpm for 15 min. Standards (0, 0.5, 1.0, 2.5, 5.0 and 10 ng/ml) and samples were incubated on acetylcholine antibody-coated 96-well flat-bottomed plates for 1 h at 37°C. The plates were washed, and then incubated with substrate A and B solutions for 15 min at 37°C. Stop solution was added and absorbances read at 450 nm using a GloMax^®^ Discover Microplate Reader (Madison, WI, USA).

### Statistical Analysis

All data are presented as means ± SDs. Data were subjected to two-way analysis of variance (ANOVA) followed by the Student’s *t*-tests or the Tukey HSD *post-hoc* test when comparing data at the same or different time points. A *P-value* <0.05 was considered statistically significant. SPSS software (SPSS for Windows, ver. 23.0; SPSS, Chicago, IL, USA) was used for all comparisons. For the behavioral tests, at least five mice per group were required to detect a between-group difference using two-way ANOVA [Cohen’s effect size f(V)  = 0 .95, α  =  0.05, 1 − β  =  0.90]. We performed behavioral tests on 40 mice; five mice in each group were sacrificed at each time point.

## Results

### Body Weight

Body weight was checked before surgery and at 2, 4, and 7 days post-operatively. A significant effect of time (pre- and post-surgery) was evident, but no effect of group (time *F* = 5.323, *P* = 0.002; group *F* = 0.563, *P* = 0.456; interaction *F* = 2.614, *P* = 0.058) was observed. Body weight decreased after surgery (pre-operative 29.87 ± 2.75 g, 2-day 2 27.37 ± 1.64 g, Tukey HSD *P* < 0.001; 4-day 28.13 ± 1.27 g, Tukey HSD *P* = 0.040) and then recovered (7-day 28.21 ± 1.68 g; Tukey HSD *P* = 0.162). Table 1 summarizes the changes in body weight over time.

**Table 1:** Differences in body weight between the two groups.

	B6	ApoE4-KI	*P*-value
Pre-operative	30.74 ± 2.91	28.28 ± 1.51	0.138
2 days^‡^	27.37 ± 1.65	27.32 ± 1.45	0.558
4 days^†^	27.96 ± 1.26	28.36 ± 1.37	0.793
7 days	28.01 ± 1.70	28.53 ± 1.96	0.717

*Each value is presented as a mean ± SD (g). Two-way ANOVA revealed a significant effect of time (pre-and post-surgery; *F* = 5.323, *P* = 0.002). ^‡^*P* < 0.01, ^†^*P* < 0.05, two-way ANOVA and the Tukey HSD *post hoc* test*.

### Behavioral Testing

#### Morris Water Maze Test

**Figure 2:**
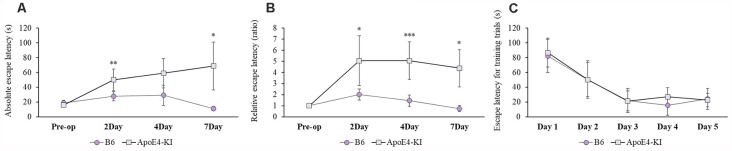
Results of Morris water maze (MWM) tests on B6 and ApoE4 transgenic knock-in (ApoE4-KI) mice. **(A)** ApoE4-KI mice showed a significantly greater mean escape latency than B6 mice at 2 and 7 days after propofol anesthesia and surgery. **(B)** ApoE4-KI mice exhibited significant increases in escape latency ratio at 2, 4, and 7 days after propofol anesthesia and surgery. **(C)** No significant difference of escape latency for training trials between the two groups. Each value a mean ± SD. *P < 0.05, **P < 0.01, ***P < 0.001.

We used the MWM test to evaluate spatial learning and memory. Two-way ANOVA revealed significant effects of time and group on both the absolute escape latency (time *F* = 3.733, *P* = 0.015; group *F* = 11.774, *P* = 0.001; interaction *F* = 2.560, *P* = 0.062) and the relative escape latency (time *F* = 3.618, *P* = 0.017; group *F* = 10.499, *P* = 0.002; interaction *F* = 1.829, *P* = 0.150). However, there was no between-groups pre-/post-operative difference in either the absolute escape latency (pre-operative 17.83 ± 2.76; 2-day 36.35 ± 12.37, Tukey HSD *P* = 0.093; 4-day 28.13 ± 16.24, Tukey HSD, *P* = 0.062; 7-day 32.75 ± 21.63, Tukey HSD *P* = 0.579) or the relative escape latency (2-day 3.17 ± 1.78, Tukey HSD *P* = 0.039; 4-day 3.00 ± 1.38, Tukey HSD *P* = 0.172; 7-day 2.12 ± 1.49, Tukey HSD *P* = 0.781). Table 2 summarizes the changes in MWM mean escape latency pre-and post-surgery. B6 and ApoE4-KI mice did not differ significantly before surgery (*P* > 0.05). However, the absolute escape latencies at 2 and 7 days after surgery differed significantly between ApoE4-KI and B6 mice (*P* < 0.05, Figure 2A). ApoE4-KI mice exhibited a significant increases in relative escape latency at all post-operative time points (2, 4, and 7 days after surgery) compared to the control mice (*P* < 0.05, Figure 2B). Two-way ANOVA did not reveal any time or group difference in terms of swimming speeds (time *F* = 0.035, *P* = 0.991; group *F* = 3.077, *P* = 0.090; interaction *F* = 0.963, *P* = 0.423) or the times spent in the four quadrants (time *F* = 0.144, *P* = 0.933; group *F* = 2.725, *P* = 0.109; interaction *F* = 0.327, *P* = 0.806). However, time, but not group, significantly affected swim path length (time *F* = 3.068, *P* = 0.043; group *F* = 0.008, *P* = 0.982; interaction *F* = 2.417, *P* = 0.086) as revealed by two-way ANOVA. On *post-hoc* analysis, significant differences were evident between the data obtained 2 days pre-operatively and those obtained 2 days post-operatively (pre-operative 552.66 ± 152.48, 2-day 1,130.10 ± 272.54; Tukey HSD *P* = 0.038), but not between the pre-operative figures and those of post-operative days 4 and 7 (4-day 986.95 ± 439.18, Tukey HSD *P* = 0.254; 7-day 608.14 ± 220.80, Tukey HSD *P* = 0.998). For training phase, no significant difference of escape latency between B6 and ApoE-KI mice (Figure 2C).

**Table 2:** Differences in Morris water maze (MWM) escape latency between the two groups—pre- and post-surgery.

	B6	ApoE4-KI	*P*-value
**A. Absolute escape latency (s)**			
Pre-operative	18.85 ± 3.16	15.98 ± 2.34	0.065
2 days	27.7 ± 6.13	50.19 ± 14.58	0.009**
4 days	29.05 ± 13.71	59.08 ± 19.84	0.288
7 days	11.16 ± 2.8	68.73 ± 32.53	0.023*
**B. Relative escape latencies (ratios)**			
Pre-operative	1 ± 0	1 ± 0	
2 days	2.00 ± 0.50	5.06 ± 2.24	0.031*
4 days	1.45 ± 0.52	5.06 ± 1.71	<0.001***
7 days	0.75 ± 0.27	4.39 ± 1.69	0.049*

A: each value is a means ± SD. Two-way ANOVA revealed significant effects of time and group (time *F* = 3.733, *P* = 0.015; group *F* = 11.774, *P* = 0.001; interaction *F* = 2.560, *P* = 0.062). *P < 0.05, **P < 0.01; two-way ANOVA and the Student *post hoct*-test. B: each value a mean ± S.D. Two-way ANOVA revealed significant effects of time and group (time *F* = 3.618, *P* = 0.017; group *F* = 10.499, *P* = 0.002; interaction *F* = 1.829, *P* = 0.150). *P < 0.05, ***P < 0.001; two-way ANOVA and the Student *post hoc* *t*-test.

#### Y-Maze Test

The Y-maze test was used to evaluate the effects of propofol anesthesia and surgery on cognitive function. Two-way ANOVA revealed no significant differences by time or group on either absolute spontaneous alternation (time *F* = 0.069, *P* = 0.976; group *F* = 0.012, *P* = 0.914; interaction *F* = 0.736, *P* = 0.535) or relative spontaneous alternation (time *F* = 0.308, *P* = 0.819; group *F* = 0.001, *P* = 0.979; interaction *F* = 0.470, *P* = 0.704). We found no difference between the pre-and post-operative data in terms of either absolute spontaneous alternation (pre-op: 54.97 ± 13.17, 2 day: 56.87 ± 22.94; Tukey HSD, *P* = 0.988; 4 day: 55.31 ± 18.83; Tukey HSD, *P* = 1.000, 7 day: 53.36 ± 28.69; Tukey HSD, *P* = 0.997) or relative spontaneous alternation (2-day 1.15 ± 0.60, Tukey HSD *P* = 0.641; 4-day 1.05 ± 0.37, Tukey HSD *P* = 0.983; 7-day 1.14 ± 0.65, Tukey HSD *P* = 0.831). Table 3 summarizes the changes in mean spontaneous alternation in the Y-maze test pre-and post-surgery. We found no significant difference between control and ApoE4-KI mice either before or after surgery. The relative spontaneous alternation after surgery also did not differ significantly between the two groups. We also analyzed the arm-entry frequency. A significant effect of time, but not group (time *F* = 18.616, *P* < 0.001; group *F* = 2.617, *P* = 0.116; interaction *F* = 0.646, *P* = 0.591) was revealed by two-way ANOVA. On *post-hoc* analysis, significant differences were evident between the pre-operative data and those obtained on post-operative days 2, 4, and 7 (pre-operative 24.00 ± 6.36; 2-day 10.83 ± 5.10, Tukey HSD *P* < 0.001; 4-day 9.75 ± 5.31, Tukey HSD *P* < 0.001; 7-day 8.67 ± 3.51, Tukey HSD *P* = 0.001).

**Table 3:** Differences in spontaneous alternation in the Y-maze test between the two groups—pre- and post-surgery.

	B6	ApoE4-KI	*P*-value
**A. Absolute spontaneous alternation (%)**			
Pre-operative	56.97 ± 3.13	49.27 ± 3.38	0.229
2 days	59.04 ± 5.6	51.81 ± 11.63	0.578
4 days	53.41 ± 8.1	57.85 ± 5.30	0.114
7 days	46.92 ± 15.38	59.8 ± 14.69	0.820
**B. Relative spontaneous alternations (ratios)**			
Pre-operative	1 ± 0	1 ± 0	
2 days	1.22 ± 0.17	0.99 ± 0.23	0.820
4 days	1.00 ± 0.15	1.12 ± 0.11	0.109
7 days	1.09 ± 0.36	1.20 ± 0.34	0.846

*A: each value is a mean ± SD. Two-way ANOVA revealed no significant effect of time or group (time *F* = 0.069, *P* = 0.976; group *F* = 0.012, *P* = 0.914 interaction *F* = 0.736, *P* = 0.535)*. B: *each value is a mean ± SD. Two-way ANOVA revealed no significant effect of time or group (time *F* = 0.308, *P* = 0.819; group *F* = 0.001, *P* = 0.979; interaction *F* = 0.470, *P* = 0.704)*.

### Histology and Biochemistry

#### Apoptosis

**Figure 3:**
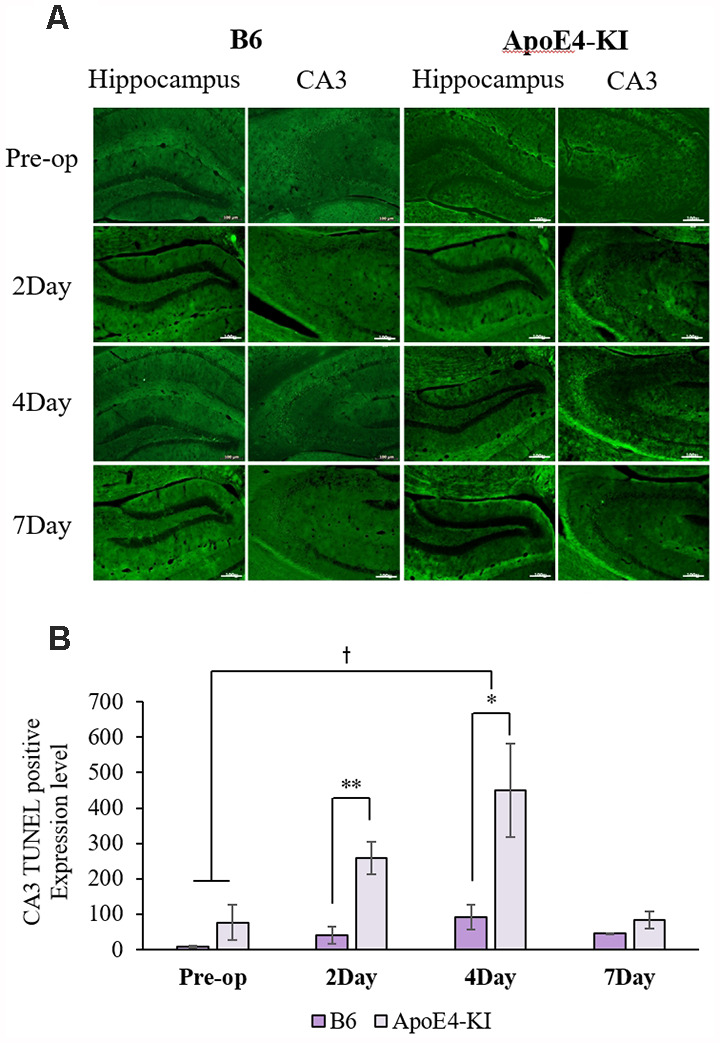
The terminal dutp nick-end labeling (TUNEL) positivities of the hippocampal CA3 regions of ApoE4-KI and control mice. **(A)** TUNEL staining (green) revealed apoptotic cells in the CA3 region (scale bar = 100 μm). **(B)** The numbers of TUNEL-positive neurons increased significantly (compared to controls) in ApoE4-KI mice on the 2nd, and 4th day after surgery, but no significant difference between the two groups was evident on day 7 after surgery. *P < 0.05, **P < 0.01. Also, significant between-group differences were apparent when the pre-operative and day 4 data were compared. ^†^*P* < 0.05. Each value is a mean ± SD.

To explore neuronal damage and the potential mechanism of cognitive decline induced by anesthesia and surgery, we measured neuronal apoptosis in the hippocampal CA3 region (Figure 3A), using the terminal dUTP nick-end labeling (TUNEL) of fragmented nuclei as viewed on an image analyzer. Brain tissues were harvested 2 days before and on the 2nd, 4th and 7th day after surgery (when the behavioral tests had concluded). Two-way ANOVA revealed significant effects of time and group on TUNEL positivity (time *F* = 3.400, *P* = 0.040; group *F* = 6.578, *P* = 0.019; interaction *F* = 1.109, *P* = 0.371). Significant differences were also apparent between the pre-operative data and those obtained on post-operative day 4 (pre-operative 51.75 ± 38.15; 4-day 319.20 ± 130.71; Tukey HSD *P* = 0.037), but not between the pre-operative data and those obtained on post-operative days 2, and 7 (2-day 176 ± 67.56, Tukey HSD *P* = 0.551; 7-day 64.75 ± 35.509, Tukey HSD *P* = 0.999). Table 4 summarizes the changes in TUNEL-positivity pre-and post-surgery. No significant difference was evident between B6 and ApoE4-KI mice before surgery. As shown in Figure 3B, the neuronal apoptosis of ApoE4-KI mice was significantly higher on post-operative days 2, and 4 compared to the control mice (*P* < 0.05). However, on post-operative day 7, there was no significant difference between the groups (Figure 3B).

**Table 4:** The TUNEL positivity levels of the two groups in the hippocampal CA3 region.

	B6	ApoE4-KI	*P*-value
Pre-operative	7.50 ± 1.77	96.00 ± 50.20	0.339
2 days	39.67 ± 24.83	257.80 ± 45.88	0.009**
4 days^†^	91.00 ± 34.82	449.00 ± 132.50	0.045*
7 days	46.50 ± 0.35	83.00 ± 24.75	0.406

Each value is a mean ± SD. Two-way ANOVA revealed significant effects of time and group (time *F* = 3.400, *P* = 0.040; group *F* = 6.578, *P* = 0.01; interaction *F* = 1.109, *P* = 0.371). *P < 0.05, **P < 0.01; two-way ANOVA and the Student *post hoct*-test; ^†^*P* < 0.05, two-way ANOVA with the Tukey HSD *post hoc* test.

#### Aβ Deposition

**Figure 4:**
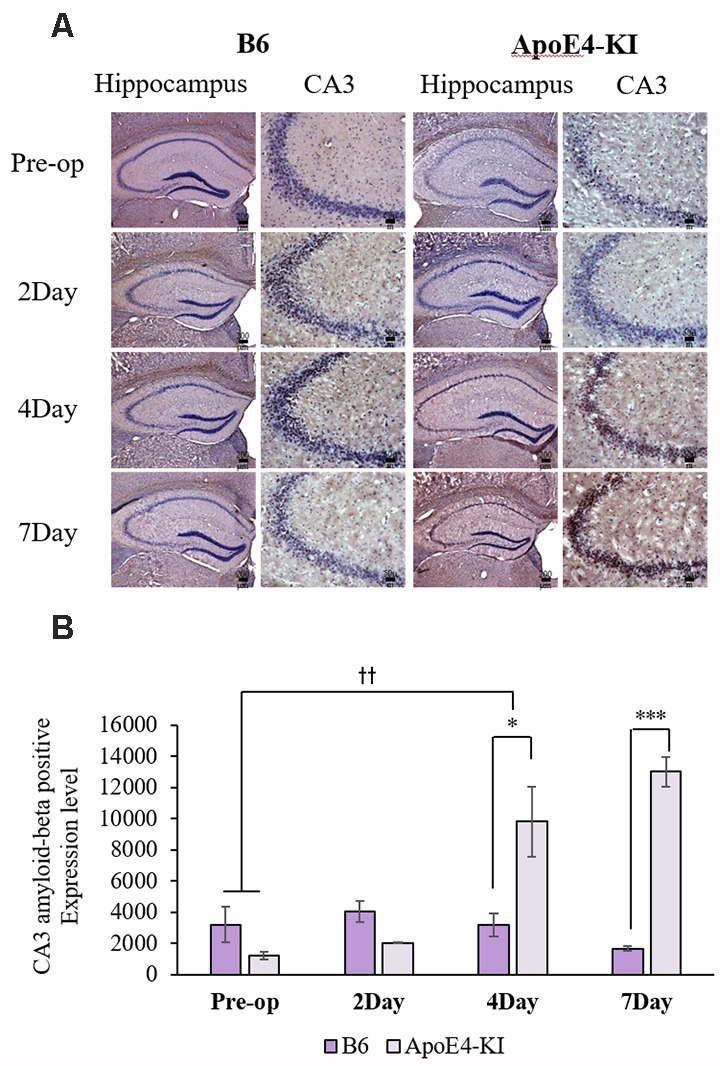
Aβ expression levels in the CA3 regions of ApoE4-KI and control mice as revealed by immunohistochemistry (IHC). **(A)** Aβ deposits stained brown in the hippocampus (scale bar = 200 μm) and also the hippocampal CA3 region (scale bar = 50 μm). **(B)** The extent of Aβ deposition did not differ significantly between the two groups pre-operatively or 2 days after surgery, but increased significantly in ApoE4-KI mice on the 4th and 7th day after surgery. *P < 0.05, ***P < 0.001. Also, the pre- and post-operative day 4 data differed significantly between the groups. ^††^*P* < 0.01. Each value is a mean ± SD.

**Figure 5:**
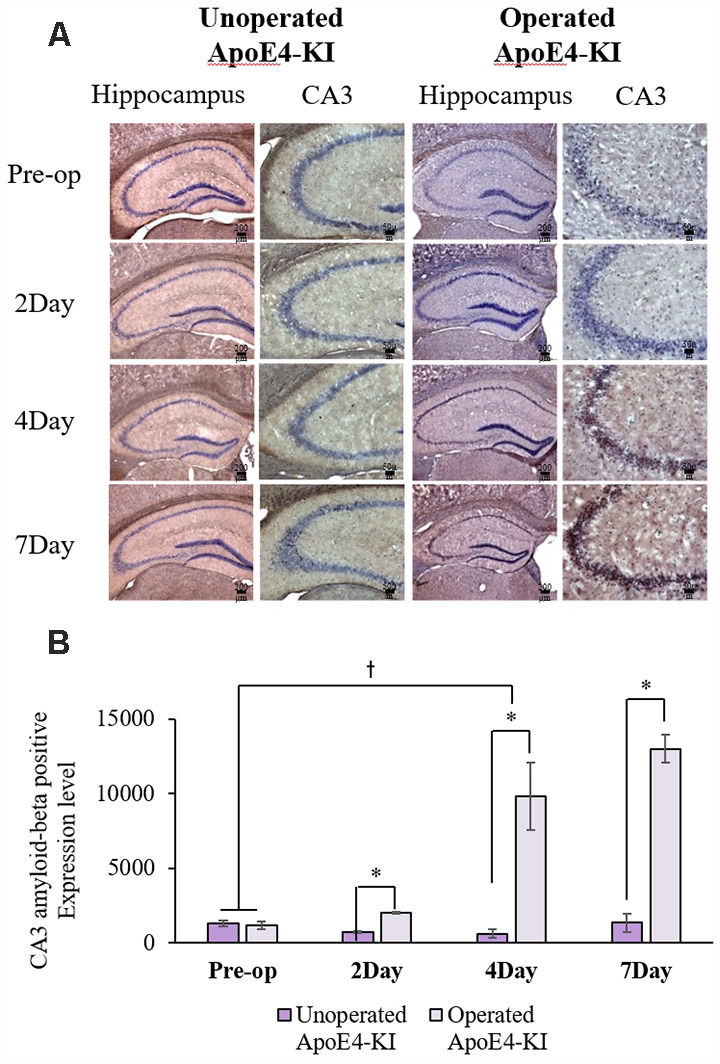
Aβ expression levels in the hippocampal CA3 regions of non-operated and operated ApoE4-KI mice as revealed by IHC. **(A)** Aβ deposits stained brown in the hippocampus (scale bar = 200 μm) and also the hippocampal CA3 region (scale bar = 50 μm). **(B)** The extent of Aβ deposition did not differ significantly between the two groups pre-operatively, but operated ApoE4-KI mice exhibited significant increases compared to non-operated age-matched ApoE4-KI mice on 2nd, 4th, and 7th day after surgery. *P < 0.05. Also, the pre-and post-operative day-4 data differed significantly between the groups ^†^*P* < 0.05. The values are means ± SDs.

We measured Aβ levels in the CA1, CA3, and dentate gyrus (DG) regions of the hippocampus. The area of Aβ deposition immunopositive deposition were measured *via* image analysis (Figure 4A). We calculated the amyloid burden, thus the percentage of the area to which antibodies to Aβ 1–42 bound. Time exerted a significant effect on all three regions, but group exerted a significant effect on only CA3 and the DG (CA1 time *F* = 6.692, *P* = 0.002; group *F* = 3.438, *P* = 0.076; interaction *F* = 1.725, *P* = 0.188; CA3 time *F* = 11.819, *P* < 0.001, group *F* = 9.163, *P* = 0.006; interaction *F* = 1.835, *P* = 0.167; DG time *F* = 7.075, *P* = 0.001; group *F* = 6.017, *P* = 0.021; interaction *F* = 2.080, *P* = 0.128) as revealed by two-way ANOVA. Immunopositive cells were measured in the CA 3 regions used in TUNEL analysis. Significant differences by time and group in terms of Aβ deposition in the hippocampal CA3 region were evident on two-way ANOVA (time *F* = 3.948, *P* = 0.019; group *F* = 7.754, *P* = 0.010; interaction *F* = 6.582, *P* = 0.002). On *post-hoc* analysis, significant increases were apparent from before operation to post-operative day 4 (pre-operative 1,997.00 ± 819.32; 4-day 7,911.69 ± 2,458.84; Tukey HSD *P* = 0.006), but not between the pre-operative and those of post-operative days 2, and 7 (2-day 3,546.75 ± 1,496.99, Tukey HSD *P* = 0.822; 7-day 4,902.86 ± 2,796.59, Tukey HSD *P* = 0.405). We found no significant differences in the CA3 Aβ expression levels of control and ApoE4-KI mice before surgery or on day 2 post-operatively (Table 5). Immunohistochemistry (IHC) revealed significantly different extents of Aβ deposition in the CA3 region in the test compared to the control groups on post-operative days 4 and 7 (*P* < 0.05, Figure 4B). When compared to non-operated age-matched ApoE4-KI mice, two-way ANOVA revealed significant effects of time and operation on Aβ deposition in the CA3 region (time *F* = 3.370, *P* = 0.043; operation *F* = 12.567, *P* = 0.002; interaction *F* = 3.423, *P* = 0.041). A significant difference was evident between the pre-operative data and those of post-operative day 4 (pre-operative 1,239.40 ± 210.34; 4-day 8,273.91 ± 2,721.12; Tukey HSD *P* = 0.005), but no differences were apparent between the pre-operative data and those of post-operative days 2 and 7 (2-day 1,376.25 ± 387.54, Tukey HSD *P* = 1.000; 7-day 7, 180.00 ± 3.424.95, Tukey HSD *P* = 0.074) on *post-hoc* analysis. We also found significant differences in terms of CA3 Aβ expression between operated and non-operated ApoE4-KI mice at all post-operative times (2, 4, and 7 days after surgery; *P* < 0.05, Figure 5).

**Table 5:** Differences in the hippocampal CA3 Aβ expression levels between the two groups as revealed by IHC.

	B6	ApoE4-KI	*P*-value
Pre-operative	3,198.50 ± 1,158.59	1,196.00 ± 264.37	0.216
2 days	4,053.67 ± 1,680.76	2,026.00 ± 142.84	0.449
4 days^‡^	3,179.67 ± 712.88	9,804.50 ± 2,266.47	0.015*
7 days	1,662.20 ± 140.38	13,004.50 ± 949.29	<0.001***

Each value is a mean ± SD. Two-way ANOVA revealed significant effects of time and group (time *F* = 3.948, *P* = 0.019; group *F* = 7.754, *P* = 0.010; interaction *F* = 6.582, *P* = 0.002). *P < 0.05, ***P < 0.001; two-way ANOVA and the Student *post hoct*-test; ^‡^*P* < 0.01, two-way ANOVA and the Tukey HSD *post hoc* test.

#### Serum Acetylcholine Levels

The serum acetylcholine levels differed significantly by group, but not time as revealed by two-way ANOVA (time *F* = 1.822, *P* = 0.154; group *F* = 5.396, *P* = 0.024; interaction *F* = 0.206, *P* = 0.854). On *post-hoc* analysis, the levels were significantly lower in ApoE4-KI mice than in B6 mice before surgery (B6 0.92 ± 0.05 (ng/ml), ApoE4-KI 0.81 ± 0.09 (ng/ml); t-test *P* = 0.043). However, there were no significant post-operative differences between the groups (2-day B6 0.93 ± 0.10 (ng/ml), ApoE4-KI 0.88 ± 0.06 (ng/ml), *t*-test *P* = 0.503; 4-day B6 1.01 ± 0.05 (ng/ml), ApoE4-KI 0.93 ± 0.04 (ng/ml), *t*-test *P* = 0.093; 7-day B6 1.06 ± 0.08 (ng/ml), ApoE4-KI 0.90 ± 0.06 (ng/ml), *t*-test *P* = 0.262).

## Discussion

We found that hippocampal cognitive function was more impaired in mice with preclinical AD than in control mice after propofol anesthesia and surgery. More Aβ accumulation was observed in the hippocampal CA3 region of the former than the latter mice, paralleled by cognitive dysfunction.

Propofol anesthesia and surgery lengthened the escape latency in the MWM test in mice with preclinical AD to a greater extent than in control mice. However, no between-group difference in the Y-maze spontaneous alternation test was apparent, reflecting the fact that the two tests assess different cognitive domains. The MWM reliably evaluates hippocampal spatial learning and memory (Vorhees and Williams, 2006). In contrast, non-hippocampal tasks such as the spontaneous alternation of the Y-maze test assess short-term memory (Yau et al., 2007); exploration is based on the choice between a novel place and a familiar place, not on learning and memory. The results imply that anesthesia/surgery impaired hippocampal-cognitive function to a greater extent in mice with preclinical AD than in age-controls.

We found that propofol anesthesia and surgery increased neuronal cell death and Aβ levels in the hippocampal CA3 region of ApoE4-KI mice to greater extents than in control mice. The neuronal apoptosis in the CA3 region of ApoE4-KI mice recovered by day 7 post-operatively, but Aβ levels continued to increase until that time. Given the increased MWM escape latency at day 7 of ApoE4-KI mice, the increased Aβ levels may have caused cognitive dysfunction. Previous studies have found correlations between Aβ accumulation and cognitive impairment in transgenic AD mice (Chiti and Dobson, 2006; Haass and Selkoe, 2007; LaFerla et al., 2007). The results imply that Aβ accumulation in the hippocampal CA3 region after surgery induced more cognitive dysfunction in mice with preclinical AD than in control mice. The CA3 region has attracted a great deal of attention in recent years, given its role in memory and its susceptibility to ischemia and neuro-degeneration. Previous studies found that the CA3 region contained extensive recurrent collaterals and an auto-association network that is critical in terms of information storage and retrieval, particularly working memory. The CA3 region is crucial in terms of acquisition and memory consolidation when mice perform the MWM task (Florian and Roullet, 2004; Kesner, 2007).

Many studies have explored possible associations between general anesthesia/surgery and POCD pathogenesis. POCD may be caused by surgery-induced neuroinflammation, elevation of brain pro-inflammatory cytokine levels, and microglial activation (Terrando et al., 2010; Wan et al., 2010). Neuroinflammation may trigger cognitive dysfunction after surgery *via* synaptic dysfunction, neuronal cell death, and microglial priming (Lyman et al., 2014); Aβ accumulation and neuroinflammation mutually enhance neurotoxicity (Yamamoto et al., 2007; Wan et al., 2010). General anesthesia *per se* (induced by inhaled anesthetics) increased Aβ production and aggregation, and tau accumulation (Run et al., 2009; Perucho et al., 2010), and inhibited the activities of cholinergic neurons in preclinical studies (Luo et al., 2020). However, clinical studies found no evidence that general anesthesia increased POCD, compared to non-general anesthesia (neuraxial, regional and local anesthesia; Mason et al., 2010; Silbert et al., 2014). Usually, anesthesia is followed by surgery. Therefore, whether the post-operative cognitive changes are attributable to the effects of anesthesia, surgery, inflammation or factors predisposing to cognitive dysfunction, remain unclear.

Both AD and POCD appear to be associated with aberrant functioning of the brain cholinergic system. in vitro studies have suggested that some anesthetics process Aβ peptide directly and/or indirectly creating a plausible link between the anesthetics and POCD (Fodale et al., 2010). In addition, anesthetics (especially inhaled agents) trigger persistent increases in the levels of AD-associated peptides; these peptides are similarly processed *in vivo* (Perouansky, 2007). Clinically relevant concentrations of isoflurane induced apoptosis of both a human neuroglioma cell line and the mouse brain (Xie et al., 2006, 2008). Desflurane, combined with hypoxia, induced Aβ production and caspase activation in both human neuroglioma and mouse brain cell lines (Zhang et al., 2008). Isoflurane induced apoptosis, in turn increasing β- and γ-secretase levels, promoting two sequential cleavages of the amyloid precursor protein to yield the Aβ peptide. Isoflurane also promoted Aβ peptide aggregation, which can cause apoptosis. Thus, a vicious cycle may be created: isoflurane induces apoptosis, Aβ is generated and aggregates, and further apoptosis follows (Xie et al., 2007). Aβ exerts acute adverse effects on multiple features of acetylcholine synthesis and release (Kar et al., 2004). In AD patients, progressive neuronal loss reduces the brain levels of acetylcholine and choline acetyltransferase, which correlate with the severity of cognitive dysfunction (Kar et al., 2004; Fodale et al., 2006). Thus, the pathomechanisms of chronic neurodegenerative disorders, including AD and POCD that involve the effects of Aβ on the central cholinergic system may overlap (Xie and Tanzi, 2006). After surgery, we found significantly increased neuronal cell death and higher Aβ levels in the CA3 region, but no significant difference in serum acetylcholine levels between preclinical AD and control mice. Serum Aβ levels spiked early in the development of an Alzheimer-like plaque pathology in an APP/PS1 transgenic mouse model; such a spike may serve as an early biomarker of AD (He et al., 2013). Another study found a direct correlation between the Aβ levels of cerebrospinal fluid (CSF) and blood in an AD mouse model (Cho et al., 2014). In a clinical study, blood Aβ levels were associated with cerebral deposition of Aβ and tau as revealed by positron emission tomography (PET), and correlated with Aβ CSF levels and plaque burden as revealed by PET (Janelidze et al., 2016; Risacher et al., 2019). However, any link between changes in blood Aβ levels and AD progression remains controversial, despite the fact that CSF Aβ levels decrease during AD development. Anesthetics such as sevoflurane reduced the blood Aβ level in aged mouse (Liang et al., 2020). We measured only blood acetylcholine levels, which is a limitation of this study. Further examination of brain tissue and CSF Aβ levels is required to confirm the association thereof with the cholinergic system.

A low pre-operative cognitive reserve may affect the link between anesthesia and long-term or persistent POCD (Bilotta et al., 2010). A previous study found that asymptomatic patients with a history of stroke were at increased risk of prolonged POCD, despite the absence of any obvious residual neurological deficits at the time of surgery (Monk et al., 2008). Another clinical study showed that pre-existing brain dysfunction was a risk factor for post-operative delirium and POCD, especially in elderly patients (Marcantonio et al., 1994). Preclinical studies found that pre-symptomatic AD transgenic mice evidenced more cognitive dysfunction after surgery than did controls (Tang et al., 2013). We found no difference in cognitive function between ApoE4-KI and control mice before anesthesia/surgery. However, the cognitive impairment of ApoE4-KI mice was more severe than that of control mice after surgery.

The effects of anesthetic agents on POCD remain controversial. A recent meta-analysis of seven studies concluded that total intravenous propofol anesthesia may reduce the risk of POCD compared to that of inhaled anesthesia, with an odds ratio of 0.52; however, the diagnostic tools used varied, as did the times of assessment and the modes of data reporting (Miller et al., 2018). Preclinical studies have suggested that general anesthetic may precipitate or exacerbate neurocognitive disorders including AD (Baranov et al., 2009). In particular, inhaled anesthetics increase the production and aggregation of Aβ peptides and induce tau phosphorylation and accumulation (Run et al., 2009; Perucho et al., 2010). As is true of AD, inhaled anesthetics trigger synaptic failure, mitochondrial dysfunction and apoptosis (Run et al., 2009). However, in another preclinical study, propofol caused less cognitive dysfunction than inhaled anesthetics, and exerted an anti-inflammatory effect (Zhang et al., 2014; Miller et al., 2018). Propofol improved cognitive function even in transgenic AD mice (Breteler et al., 1991; Shao et al., 2014; Nie et al., 2020), in contrast to our present findings. The differences may be attributable to the various research methods employed; the AD mouse model used [3xTgAD (Mardini et al., 2017); APPswe (Yang et al., 2014; Zhang et al., 2014); the age of the animals; and the dose, injection method, and duration of propofol anesthesia [250 mg/kg intraperitoneally over 25 min (Mardini et al., 2017), 50 mg/kg intraperitoneally over 30 min (Shao et al., 2014; Nie et al., 2020)]. In addition, earlier studies used the classical mouse model, which is clinically relevant to only early-onset familial AD (Borchelt et al., 1996). We used the APOE-KI mouse model, of late-onset sporadic AD that is common in clinical settings (Campion et al., 1999; King, 2018). Further studies are needed to clarify the effects of anesthetics on POCD.

Our findings may have clinical implications. Patients with mild cognitive impairment (MCI) or preclinical AD may be at greater risk of POCD than others, consistent with the clinical observation that elderly patients with higher brain Aβ levels (Fukumoto et al., 2004) and carriers of the gene encoding apolipoprotein E4 (Cao et al., 2014), are more likely to develop POCD (Moller et al., 1998).

Our study had several limitations. First, although we performed the MWM and Y-maze tests, only the MWM test yielded meaningful results. The hippocampus and other brain areas are involved in cognitive function. Future studies should include other tests, such as a novel-object-recognition test (NOR), to further assess whether anesthesia/surgery impairs cognitive function in mice with preclinical AD. NOR is widely used as a measure of recognition memory in AD mice, and serves as a model of episodic-like memory in humans (Zhang et al., 2012; Lueptow, 2017). Second, we lacked data later than 1 week after surgery. Previous POCD studies also lacked such data (Rosczyk et al., 2008; Fidalgo et al., 2011). However, the cognitive decline and Aβ levels of ApoE4-KI mice continued to increase until 7 days post-operatively. This may reflect either slow recovery from cognitive impairment or progression to AD and cognitive dysfunction. Third, we used propofol only to induce general anesthesia; we did not explore any association of POCD with inhalant anesthesia. Longer-term follow-up and evaluation of inhaled anesthetics are required. Also, we used only male mice. Most behavioral studies use only males to avoid variability caused by the female estrous cycle (Rosczyk et al., 2008). Many epidemiological and clinical studies have identified sex differences in AD susceptibility among ApoE4 carriers, although the mechanism in play remains unclear. ApoE4 KI mouse exhibits a sexual difference in terms of the numbers of hilar GABAergic interneurons evident as AD develops (Orthofer et al., 2020). Also, we retro-orbitally injected propofol. In mice, intravenous injections are commonly delivered into the lateral tail vein. This is sometimes difficult and may stress the mice. A previous experiment using transgenic mice found that retro-orbital injection was the less stressful of the two methods (Steel et al., 2008). Retro-orbital injection is an acceptable alternative to tail-vein injection, being technically less challenging and allowing repeat injections (Yardeni et al., 2011). We wished to maintain anesthesia for 2 h, hence our use of the retro-orbital route.

Propofol anesthesia/surgery caused persistent changes in cognition and pathological changes in the hippocampus of pre-symptomatic but vulnerable AD mice. It would be interesting to explore whether patients with MCI or preclinical AD are at greater risk of POCD. It is difficult to diagnose preclinical AD; however, in patients with risk factors for AD, such as a familial history, genetic predisposition, and/or a history of stroke, diabetes, and/or MCI, clinicians should be careful when planning anesthesia and surgery.

## Data Availability Statement

The original contributions presented in the study are included in the article, further inquiries can be directed to the corresponding author.

## Ethics Statement

The animal study was reviewed and approved by the institutional Animal Care and Use Committees of Hallym University.

## Author Contributions

J-HS: conception and design of the study. J-HK, HJ, YL, and J-HS: acquisition of data, analysis and interpretation of data. J-HK, HJ, and J-HS: writing of the manuscript. All authors contributed to the article and approved the submitted version.

## Conflict of Interest

The authors declare that the research was conducted in the absence of any commercial or financial relationships that could be construed as a potential conflict of interest.

## Abbreviations

POCD: postoperative cognitive dysfunction
AD: Alzheimer’s disease
MWM: Morris water maze
Aβ: amyloid-beta
ApoE4: apolipoprotein E4 allele
ApoE4-KI: ApoE4 transgenic knock-in
OCT: optimal cutting temperature
H&E: hematoxylin and eosin
TUNEL: terminal-deoxynucleotidyl transferase mediated nick end labeling
PBS: phosphate-buffered saline
IHC: immunohistochemistry
CSF: cerebrospinal fluid
PET: positron emission tomography
MCI: mild cognitive impairment
NOR: novel-object-recognition test.
